# Twist Expression in Circulating Hepatocellular Carcinoma Cells Predicts Metastasis and Prognoses

**DOI:** 10.1155/2018/3789613

**Published:** 2018-06-26

**Authors:** Liang-Chun Yin, Zhen-Chao Luo, Yan-Xin Gao, Yang Li, Qing Peng, Yi Gao

**Affiliations:** ^1^Department of Hepatobiliary Surgery II, Guangdong Provincial Research Center for Artificial Organ and Tissue Engineering, Guangzhou Clinical Research and Transformation Center for Artificial Liver, Institute of Regenerative Medicine, Zhujiang Hospital, Southern Medical University, Guangzhou, Guangdong 510280, China; ^2^Department of General Surgery, The Third Affiliated Hospital of Southern Medical University, Guangzhou, Guangdong 510630, China; ^3^State Key Laboratory of Organ Failure Research, Southern Medical University, Guangzhou, Guangdong 510282, China

## Abstract

Hepatocellular carcinoma (HCC) is one of the leading malignancies worldwide. Enumeration of circulating tumor cells (CTCs) has been demonstrated to be a prognostic indicator in HCC. Twist plays a critical role in metastasis and has been proposed as a biomarker for epithelial-mesenchymal transition (EMT). However, links between the expression of Twist in CTCs and HCC clinical parameters are still unclear. This study aims to evaluate the relationship between Twist expression in CTCs and clinicohistopathological risk factors of HCC. Between June 2015 and July 2017, 80 HCC patients and 10 healthy volunteers were enrolled in this study. CTCs were isolated and analyzed by the optimized CanPatrol™ CTC-enrichment technique. Our analysis showed that Twist+ CTCs were detected in 54 of the 80 (67.5%) HCC patients. The positive ratios of Twist+ CTCs correlated with portal vein tumor thrombi, TNM staging, AFP, cirrhosis, tumor number, tumor size, and microvascular invasion. Meanwhile, the follow-up results of the 33 HCC patients who underwent hepatectomy showed that the positive ratios of Twist+ CTCs were closely correlated with the rate of metastasis or recurrence and the mortality rate. The ROC curve analyses suggested that the prognostic evaluation of Twist+ CTCs outperforms CTCs alone. Twist+ CTCs showed higher expression in Glypican-3 protein. In conclusion, Twist expression in CTCs could serve as a biomarker for evaluating HCC metastasis and prognosis.

## 1. Introduction

Hepatocellular carcinoma (HCC) is one of the leading malignancies worldwide. In men, it is the fifth most frequently diagnosed cancer and is the second leading cause of cancer-related deaths worldwide. In women, it is the seventh most frequently diagnosed cancer and is the sixth leading cause of cancer-related deaths worldwide. Although upgrades in systemic perioperative treatment and diagnostic methods have contributed to a decrease in its mortality and an increase in the early diagnosis of HCC over the past few decades, the 5-year recurrence rate after curative treatment remains high (70%) [1]. About 740,000 new HCC cases and 690,000 cancer-related deaths occur worldwide per year, and approximately half of these cases and deaths occur in China [2, 3]. HCC is related to a high frequency for vascular invasion, which results in poor cancer prognosis [4].

Tumor progression and metastasis are complex processes that remain unclear. A number of studies indicate that the mechanism termed epithelial-mesenchymal transition (EMT), a physiological process first observed in embryonic development [5], plays a crucial role during cancer progression and metastasis formation [6]. EMT is a cellular process during which epithelial cells lose cell-cell contacts and cell polarity, downregulate epithelial-associated genes, acquire mesenchymal features, and undergo major changes in their cytoskeletons [7–9]. Circulating tumor cells (CTCs), which are trace cells shed into the bloodstream from primary or metastatic tumors, circulate in the bloodstream, constituting seeds for distant secondary metastasis [10]. EMT of individual cells within a primary tumor leads to their intravasation into the peripheral circulation. Survival of such CTCs within the bloodstream extravasates to distant organs; then they undergo a reverse process termed mesenchymal-to-epithelial transition (MET) and establishment of new tumors [11].

In 1869, CTCs were first described by Thomas Ashworth [12], but only recently have the enumeration and characterization of CTCs been shown to be clinically useful as an independent prognostic and treatment efficacy biomarker in epithelial malignancies. Detection and characterization of CTCs can provide both the phenotype and genotype of primary tumors. Thus, CTCs may serve as a “liquid biopsy” for metastatic tumors. In comparison to traditional metastatic tissue biopsy, isolation of CTCs as a “liquid biopsy” provides many unique advantages: collection of peripheral blood is easy to perform; the procedure is rapid, noninvasive, and cost-effective and allows for serial real-time monitoring. To date, extensive studies have demonstrated that CTCs are useful prognostic biomarkers in different types of cancer, including metastatic breast [13], colorectal [14], and prostate [15] cancers. Based on these clinical studies, the US Food and Drug Administration approved Veridex's CELLSEARCH® technology for CTC enrichment and enumeration for the above indicated cancers. In addition, several studies have reported that the presence of CTCs in peripheral blood is intimately associated with metastasis and prognosis of HCC patients [16].

EMT is thought to be controlled by a family of genes. To date, a large body of research has shown that many transcription factors (e.g., Snail, Slug, ZEB1, and FOXC2) have been identified as capable of inducing the EMT process [17–20]. Lately, the highly conserved helix-loop-helix (bHLH) transcription factor (TF) Twist has been identified as a regulator of EMT [20]. Twist plays a critical role in metastasis and has been proposed as a biomarker for EMT [20]. The expression of Twist in HCC patients has been connected to poor prognoses [21]. However, links between the expression of Twist in CTCs and HCC clinical parameters are still unclear.

Therefore, for the first time, this study applied the advanced CanPatrol CTC-enrichment technique to detect and analyze Twist+ CTCs in HCC patients. We will primarily discuss whether EMT-inducing TF Twist is expressed in CTCs and whether its expression levels may act as a related prognostic factor in HCC patients.

## 2. Materials and Methods

### 2.1. Patients

Between June 2015 and July 2017, a total of 80 HCC patients in our institution and 10 healthy volunteers were enrolled in this study. 41 HCC patients underwent hepatectomy, and 33 of whom had completed a 1-year period of follow-up. 18 HCC patients underwent transcatheter arterial chemoembolization (TACE), and 14 of whom had completed a 1-year period of follow-up. The remaining 21 patients were untreated. The subject inclusion criteria were as follows: older than 18 years, histopathologically diagnosed using surgically resected specimens or liver biopsies, had not received preoperative chemotherapy or radiation therapy, and no other inflammatory disease or malignant tumor history. Clinicopathological characteristics are summarized in Table 1. TNM stage was determined according to the American Joint Committee on Cancer TNM Staging for Liver Tumors (7th edition, 2010, ISBN 0387884408). The tumor differentiation stage was defined according to the Edmondson-Steiner grading system. Written informed consent statements were obtained from all HCC patients and healthy volunteers involved in this study. The study protocol was approved by the Ethics and Scientific Committees of our institution.

### 2.2. Follow-Up and Prognosis

To date, we have completed a 1-year period of follow-up of the 47 HCC patients who underwent hepatectomy or TACE. They were followed up by telephone or outpatient service every month. The postoperative survival time of patient was defined as the time interval between surgery and death or the last follow-up. The surveillance information includes AFP, radiographic imaging, or CTCs. The postoperative recurrence standards of patients who underwent hepatectomy were as follows: intrahepatic new tumor and postoperative AFP that reduced to normal and then increased again. The postoperative metastasis standard of patients who underwent hepatectomy was as follows: extrahepatic new tumor. The assessment of TACE was estimated according to the mRECIST criteria (Supplementary 1).

### 2.3. Blood Sample Collection

Peripheral blood samples were obtained from selected patients at admission or 1-7 days before hepatectomy or TACE. After discarding the first 2 ml of drawn peripheral blood to avoid potential skin cell contamination, 5 ml peripheral blood samples from our selected patients were collected into EDTA (Ethylenediaminetetraacetic Acid) tubes by venipuncture. Samples were maintained at 4°C and were analyzed with the CanPatrol System (SurExam BioTech, Guangzhou, China) [22] within 4 h after collection. In addition, 5 ml blood samples from 10 healthy volunteers were used as negative controls or for spiking experiments.

### 2.4. Isolation of CTCs Using the CanPatrol CTC Filtration System

The CanPatrol CTC filtration system (SurExam BioTech, Guangzhou, China) was used for the isolation of CTCs. This system included a filtration tube containing a calibrated membrane with 8 *μ*m diameter pores (Millipore, Billerica, MA, USA), a manifold vacuum plate with valve settings (Millipore), an E-Z 96 vacuum manifold (Omega, Norcross, GA, USA), and a vacuum pump (AUTOSCIENCE, Tianjin, China). The samples were centrifuged to collect cellular pellets. Before filtration, red blood cell lysis buffer that consisted of 0.1 mM EDTA, 10 mM KHCO_3_, and 154 mM NH_4_Cl (all from Sigma, St. Louis, MO, USA) in deionized water was applied to remove erythrocytes. The supernatant was discarded; then 5 ml PBS (Sigma) with 4% formaldehyde (Sigma) was used to resuspend the remaining cell pellets for 5 min. The cell suspension was transferred to the filtration tube under vacuum pressure (0.08 MPa). The CTCs, which are larger than blood cells, were ultimately retained on the filter, and the blood cells passed through the filter pores. Then the cells retained on the filter were fixed by washing with 2% formaldehyde solution.

### 2.5. Identification and Characterization of CTCs Using RNA In Situ Hybridization (RNA-ISH)

The RNA-ISH method, which is based upon branched deoxyribonucleic acid (bDNA) signal amplification technology, was used to detect and classify CTCs. The sensitivity of bDNA signal amplification technology, which uses a multistep nucleic acid hybridization platform, is accomplished by signal amplification of a bDNA probe after direct binding of multiple specific capture probes to target sequences [23]. Sequences of epithelial biomarkers (EpCAM and CK8/18/19), a mesenchymal biomarker (Twist) and a leukocyte biomarker (CD45), which were used to distinguish CTCs, are listed in Supplementary 2. The details of the hybridization assay procedure have been published by Yu et al. [24].

Three groups of nucleic acid probes were used in this study to identify the expression levels of epithelial and mesenchymal genes in CTCs by multiplex RNA-ISH assay. Group 1 probes comprised four pooled epithelial transcripts (EpCAM and CK8/18/19). Group 2 probes had a mesenchymal transcript (Twist). Group 3 probes had a CD45 transcript, which was used to discriminate CTCs from leukocytes. All the sequences of the capture probes were synthesized by Invitrogen (Invitrogen, Shanghai, China).

The cells retained on the filter membrane of the 24-well plate (Corning, NY, USA) were treated with protease (Qiagen, Hilden, Germany) and then subjected to serial hybridization reactions with the capture probes described above. After incubation at 42°C for 2 h, the unbound probes were removed by washing three times with 1,000 *μ*l of wash buffer (Sigma). Finally, we used the nucleic acid dye 4′,6-diamidino-2-phenylindole (DAPI) (Sigma) to stain cell nuclei. The CTCs were analyzed with an automated imaging fluorescent microscope Axio Imager Z2 (Carl Zeiss Meditec AG, Germany). The red dots representing fluorescent signals observed in the cells indicated epithelial biomarker expression. The green dots representing fluorescent signals observed in the cells indicated the mesenchymal biomarker for Twist expression. The bright white dots representing fluorescent signals observed in the cells indicated CD45 expression, a marker for leukocytes.

### 2.6. Spiking Experiments

To demonstrate the sensitivity and linearity of CTC recovery using this technique, the human hepatocarcinoma (HepG2) cell lines (10, 50, 100, and 200 HepG2 cells, ATCC, HB 8065, derived from a human HCC) were spiked into 5 ml of blood collected from healthy volunteers eight times to validate the sensitivity and linearity of this technique.

### 2.7. Statistical Analysis

The chi-squared test and Fisher's exact test were used in testing for association between two categorical variables. Spearman's rank correlation test was applied when target categorical variable was ordinal. *P* values < 0.05 were considered statistically significant. All statistical tests were two-sided. The prognostic evaluations between Twist+ CTCs and CTCs were compared by receiver operator characteristic (ROC) curves analyses, and the area under the curve (AUC) was calculated. All statistical processing was performed using the Statistical Package for Social Sciences, version 22.0 (SPSS, Inc., Chicago, IL, USA).

## 3. Results

### 3.1. Patients' Characteristics

In total, 80 patients with HCC and 10 healthy volunteers were enrolled in this study. The clinical characteristics of HCC patients are summarized in Table 1.

### 3.2. EpCAM, CK8/18/19, and Twist Expression in HepG2 Cells and Blood Leukocytes from Healthy Donors

Probes were validated using the HepG2 cell line to confirm EpCAM, CK8/18/19, and Twist expression in CTCs. EpCAM, CK8/18/19, and Twist expression were also measured in leukocytes from healthy volunteers. Among these biomarkers, CD45 was expressed in leukocytes but not in tumor cells. Therefore, CTCs were defined as epithelial biomarkers (EpCAM and CK8/18/19) and/or the mesenchymal biomarker- (Twist-) positive CD45^−^DAPI^+^ intact cells. Twist+ CTCs were defined as Twist-positive CD45^−^DAPI^+^ intact cells. Leukocytes were defined as CD45^+^DAPI^+^ cells (Supplementary 3).

### 3.3. CTC Detection Efficiency

**Figure 1:**
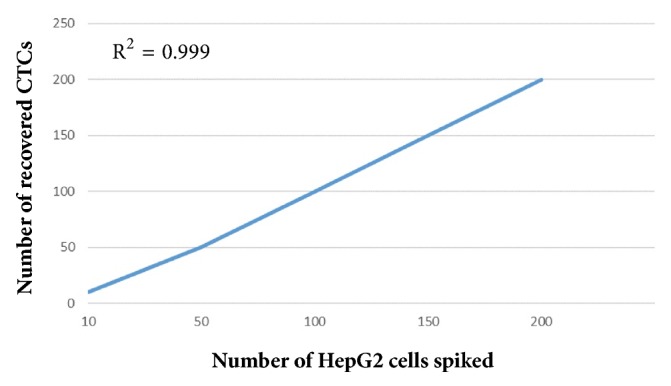
Regression analysis of the recovered CTCs by the CanPatrol CTC-enrichment technique against the number of HepG2 cells spiked.** Note.** HepG2: human hepatocarcinoma; CTC: circulating tumor cell.

The spiking experiments demonstrated that detection of CTCs proceeded in a linear fashion (*R*^2^ = 0.999). The average tumor cell recovery was at least 80% (Figure 1).

### 3.4. Expression of CTCs in the 80 HCC Patients

**Figure 2:**
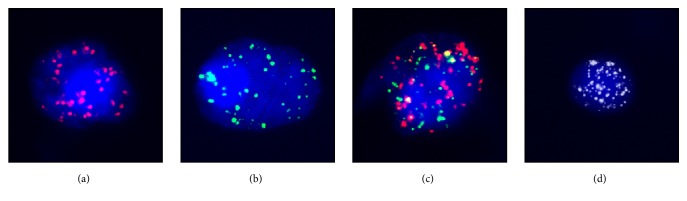
CTCs detected in a blood sample from a HCC patient under the automated imaging fluorescent microscope.** Note.** (a) Representative images of CTCs stained for epithelial biomarker EpCAM and CK8/18/19 (red dots). (b) Representative images of CTCs stained for the mesenchymal biomarker Twist (green dots). (c) Representative images of CTCs stained for epithelial biomarker EpCAM, CK8/18/19 (red dots), and the mesenchymal biomarker Twist (green dots). (d) Representative images of negative control and leukocytes stained for CD45 expression (bright white fluorescence). The cells were analyzed using a 100x oil objective. CTCs: circulating tumor cells; HCC: hepatocellular carcinoma; EpCAM: epithelial cell adhesion molecule; CK: cytokeratin.

The characteristics of CTCs isolated from HCC patients included a larger cell size with intact nuclei, irregular shape, and a high nuclear to cytoplasmic ratio (Figure 2). The CTCs were positively stained for DAPI, and different fluorescence signals of epithelial biomarkers (EpCAM and CK8/18/19) and the mesenchymal biomarker (Twist) were measured to distinguish types of CTCs. Leukocytes were collected as a negative control.

The results showed that CTCs were detected in blood samples obtained from 62 of the 80 (77.5%) HCC patients. The number of CTCs detected in these patients ranged from 0 to 52, and the average number of CTCs was 14.04 (detailed results are shown in Table 1). No CTCs were detected in any of the blood samples from healthy volunteers.

### 3.5. CTCs/Twist+ CTCs and Clinicohistopathological Risk Factors of the 80 HCC Patients

We explored the relationship between CTCs and clinicohistopathological risk factors of the 80 HCC patients. The chi-squared test and Fisher's exact test indicated that there was a correlation between the positive ratios of CTCs and portal vein tumor thrombi. The positive ratios of CTCs were significantly higher in patients with portal vein tumor thrombi (46/46) than in patients without portal vein tumor thrombi (16/34; *χ*^2^ = 4.267, *P* = 0.039; Table 2). In addition, tumor number was higher in CTC+ groups than in CTC− groups (*χ*^2^ = 4.219, *P* = 0.040; Table 2). However, there were no differences between CTC+ and CTC− groups in other clinicopathological factors, such as age, gender, Child-Pugh class, tumor size, TNM staging, cirrhosis, or serum AFP level (Table 2).

Using RNA-ISH assays, we identified Twist+ CTCs according to the EMT markers used in this study. We explored the relationship between Twist+ CTCs and clinicohistopathological risk factors of the 80 HCC patients. The relationships of Twist+ CTCs to various clinicopathological characteristics are shown in Table 2. Positive ratios of Twist+ CTCs were detected in 54 of the 80 (67.5%) HCC patients. The number of Twist+ CTCs detected in these patients ranged from 0 to 16, and the average number of Twist+ CTCs was 3.49 (detailed results are shown in Table 1). The positive ratios of Twist+ CTCs were significantly higher in patients with portal vein tumor thrombi (46/46) than in patients without portal vein tumor thrombi (8/34; *χ*^2^ = 11.483, *P* = 0.001; Table 2). The positive ratios of Twist+ CTCs highly correlated with TNM staging, with 0.0% (0/9) positivity in stage I and 100.0% (10/10) positivity in stage IV (*χ*^2^ = 8.435, *P* = 0.038; Table 2). The positive ratios of Twist+ CTCs increased in the metastatic stages of HCC. In addition, there was a high correlation between the positive ratios of Twist+ CTCs and tumor number (*χ*^2^ = 12.321, *P* < 0.001; Table 2). In addition, there was a correlation between the positive ratios of Twist+ CTCs and tumor size (*χ*^2^ = 8.021, *P* = 0.018; Table 2). There was a high correlation between the positive ratios of Twist+ CTCs and cirrhosis (*χ*^2^ = 11.091, *P* = 0.001; Table 2). There was a correlation between the positive ratios of Twist+ CTCs and AFP level (*χ*^2^ = 8.724, *P* = 0.033; Table 2). However, there were no differences between the positive ratios of Twist+ CTCs and other clinical parameters, including age, gender, or Child-Pugh class (Table 2).

### 3.6. Analysis of Prognostic Evaluation between CTCs and Twist+ CTCs

**Figure 3:**
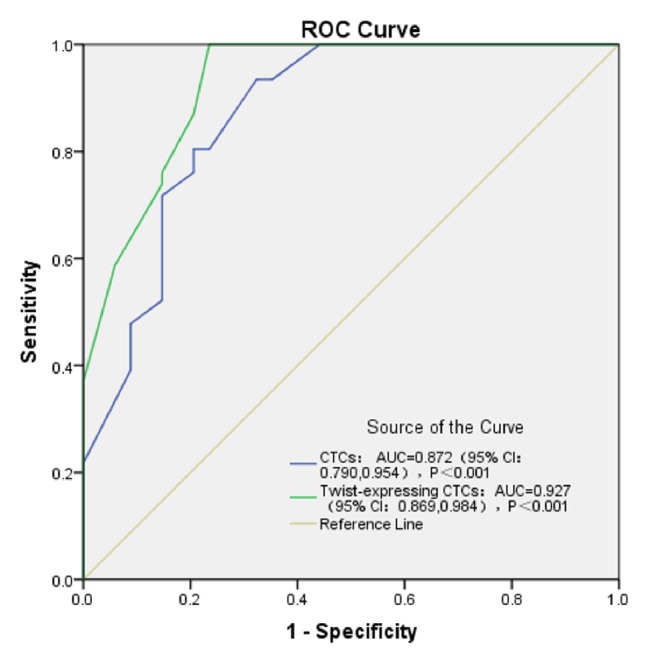
ROC curve for CTCs and Twist+ CTCs in predicting portal vein tumor thrombi.

**Figure 4:**
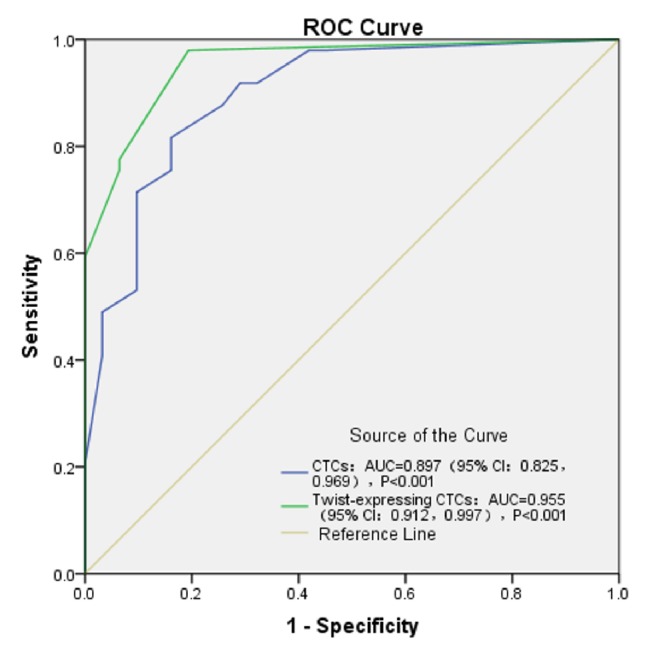
ROC curve for CTCs and Twist+ CTCs in predicting tumor number.

To compare the prognostic evaluations between CTCs and Twist+ CTCs, we performed ROC curve analyses.  Figure 3 shows the ROC curve for CTCs and Twist+ CTCs in predicting portal vein tumor thrombi, in which the assay demonstrated an area under the curve (AUC) of 0.872 and 0.927, respectively.  Figure 4 shows the ROC curve for CTCs and Twist+ CTCs in predicting tumor number, in which the assay demonstrated an AUC of 0.897 and 0.955, respectively. The ROC curve analyses suggested that the prognostic evaluation of Twist+ CTCs outperforms CTCs alone.

### 3.7. CTCs/Twist+ CTCs and Histopathological Risk Factors of the 41 HCC Patients Who Underwent Hepatectomy

To explore the relationship between CTCs and histopathologic risk factors of the 41 HCC patients who underwent hepatectomy, histopathological features (including microvascular invasion and tumor differentiation stage) were evaluated (Supplementary 4). Microvascular invasion (MVI) was identified in 19 patients (19/41; Supplementary 4). Our results showed that no association was found between the positive ratios of CTCs and the presence of MVI (*χ*^2^ = 4.143, *P* = 0.052; Table 3) and no association was found between the positive ratios of CTCs and tumor differentiation stage (*χ*^2^ = 3.425, *P* = 0.232; Table 3).

Meanwhile, our results showed that the positive ratios of Twist+ CTCs correlated with the presence of MVI (*χ*^2^ = 9.616, *P* = 0.004; Table 3). However, no association was found between the positive ratios of Twist+ CTCs and tumor differentiation stage (*χ*^2^ = 4.909, *P* = 0.089; Table 3)

### 3.8. Immunohistochemistry of Samples of the 41 HCC Patients Who Underwent Hepatectomy

**Figure 5:**
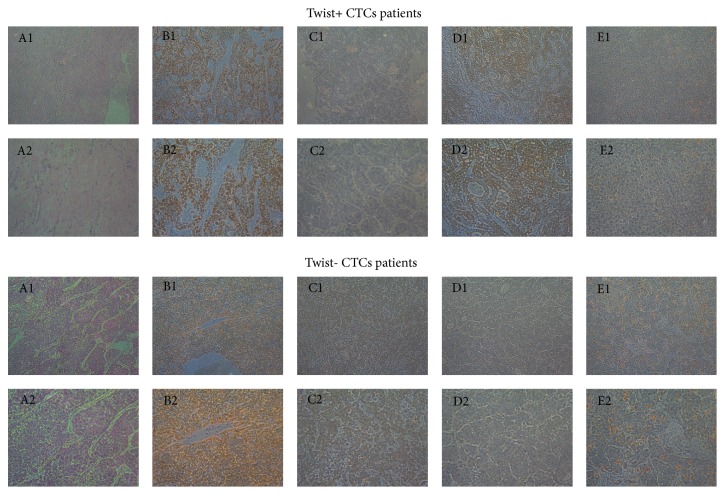
HE, HBC-AG, CK19, Glypican-3, and Ki-67 expression in surgical specimens of 41 HCC patients who underwent hepatectomy.** Note.** A1: HE, 100x; A2: HE, 200x; B1: HBC-AG, 100x; B2: HBC-AG, 200x; C1: CK19, 100x; C2: CK19, 200x; D1: Glypican-3, 100x; D2: Glypican-3, 200x; E1: Ki-67, 100x; E2: Ki-67, 200x.

HBC-AG, CK19, Glypican-3, and Ki-67 protein expressions were examined in the samples of the 41 HCC patients who underwent hepatectomy. Glypican-3 protein was expressed in 25 of the 41 HCC patients who underwent hepatectomy. It was shown that the positive ratios of Twist+ CTCs highly correlated with Glypican-3 protein (*χ*^2^ = 8.050, *P* = 0.005). However, there were no differences between the positive ratios of Twist+ CTCs and other proteins (Table 4 and Figure 5).

### 3.9. CTCs/Twist+ CTCs and Therapeutic Response of 4 HCC Patients Who Underwent Hepatectomy or TACE

In this study, we observed the number variation of CTCs and Twist+ CTCs in 4 HCC patients between 1 and 7 days before hepatectomy or TACE and 2 months after hepatectomy or TACE. The results showed a decrease in CTCs and Twist+ CTCs count after therapy (Supplementary 5).

### 3.10. CTCs/Twist+ CTCs and Prognosis of the 33 HCC Patients Who Underwent Hepatectomy

To date, we have completed a 1-year period of follow-up of the 33 HCC patients who underwent hepatectomy: 23 patients with metastasis or recurrence (23/33) and 21 patients with death (21/33). Our results showed that the rate of metastasis or recurrence of the patients who underwent hepatectomy was higher in the CTC+ group (20/25) than in the CTC− group (3/8; *χ*^2^ = 5.183, *P* = 0.036; Table 5). The mortality rate of the patients who underwent hepatectomy was also higher in the CTC+ group (19/25) than in the CTC− group (2/8; *χ*^2^ = 6.812, *P* = 0.015; Table 5).

**Figure 6:**
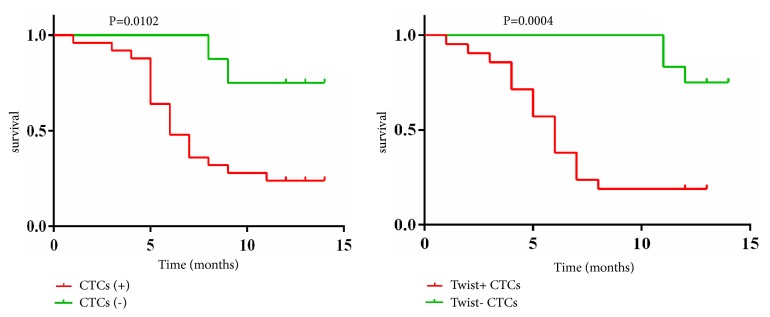
Log-rank test of survival analysis of the 33 HCC patients who underwent hepatectomy. CTCs: circulating tumor cells.

Meanwhile, the results showed that the rate of metastasis or recurrence of the patients who underwent hepatectomy was significantly higher in the Twist+ CTC group (19/21) than in the Twist− CTC group (4/12; *χ*^2^ = 11.806, *P* = 0.001; Table 5). The mortality rate of the patients who underwent hepatectomy was also significantly higher in the Twist+ CTC group (18/21) than in the Twist− CTC group (3/12; *χ*^2^ = 12.165, *P* = 0.001; Table 5).  Figure 6 shows the log-rank test of survival analysis of the 33 HCC patients who underwent hepatectomy.

### 3.11. CTCs/Twist+ CTCs and Prognosis of the 14 HCC Patients Who Underwent TACE

To date, we have completed a 1-year period of follow-up of the 14 HCC patients who underwent TACE: 9 patients with progressive disease (PD) (9/14) and 8 patients with death (8/14). Our results showed that the PD rate of the patients who underwent TACE was higher in the CTC+ group (7/9) than in the CTC− group (2/5; *χ*^2^ = 1.988, *P* = 0.266; Table 6). However, the difference was not statistically significant. The mortality rate of the patients who underwent TACE was also higher in the CTC+ group (6/9) than in the CTC− group (2/5; *χ*^2^ = 0.933, *P* = 0.580; Table 6). However, the difference was also not statistically significant.

**Figure 7:**
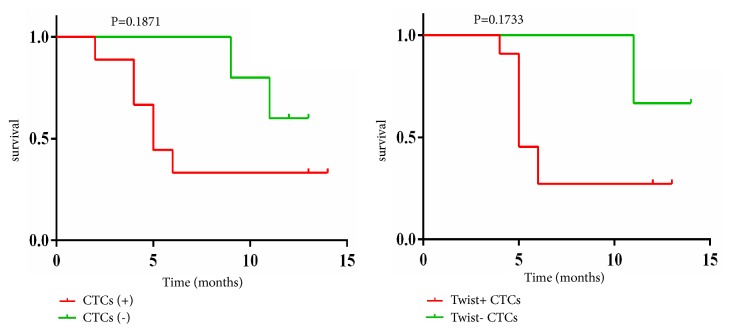
Log-rank test of survival analysis of the 14 HCC patients who underwent TACE. CTCs: circulating tumor cells; TACE: transcatheter arterial chemoembolization.

Meanwhile, the results showed that the PD rate of the patients who underwent TACE was higher in the Twist+ CTC group (7/8) than in the Twist− CTC group (2/6; *χ*^2^ = 4.381, *P* = 0.091; Table 6). However, the difference was not statistically significant. The mortality rate of the patients who underwent TACE was also higher in the Twist+ CTC group (6/8) than in the Twist− CTC group (2/6; *χ*^2^ = 2.431, *P* = 0.277; Table 6). However, the difference was also not statistically significant.  Figure 7 shows the log-rank test of survival analysis of the 14 HCC patients who underwent TACE.

## 4. Discussion

Early diagnosis and treatment of HCC may prevent its subsequent metastasis. Currently, the prognostic information used includes patient history, physical examinations, blood testing, and radiographic imaging. Although these examinations may be helpful, they are often difficult to follow up, expensive, inconvenient, and inaccurate. In addition, therapeutic efficacy is significantly hampered by the genetic and phenotypic alterations of cancer cells. However, traditional multiple regular metastatic tissue biopsies may be unavailable or infeasible. Thus, survival predictions for cancer patients are very difficult for clinicians to accurately assess. CTCs, as a source for longitudinal molecular analysis of cancer, can provide necessary molecular information for clinicians for improved prognostication and treatment strategy. Isolation and analysis of CTCs in peripheral blood may detect cancer progression earlier than radiographic examinations and can be applied to early diagnosis of metastasis [13]. A number of clinical studies of patients with different metastatic cancers have shown that increased CTC numbers are associated with poor prognosis [13, 25]. Since CTCs frequently can be detected and analyzed, CTC enumeration may also be suggested as an accurate and rapid surrogate biomarker or “liquid biopsy” to evaluate the prognosis and therapeutic efficacy of cancer during the entire course of the disease [26]. The success of Veridex's CELLSEARCH technology proves that enumeration and characterization of CTCs are indeed a clinical biomarker for cancer. Thus, CTCs may facilitate estimation of a prognosis, real-time monitoring of therapies, early relapse detection, and clinical study of metastasis mechanisms.

Many studies have shown that Twist overexpression predicts a poor prognosis for melanoma, breast cancer, and HCC [27–29]. Previous studies demonstrate that Twist overexpression in CTCs correlates with cancer metastasis [30]. So far, only a few data have been published underlying the clinical relevance of Twist+ CTCs in the blood of HCC patients. Accordingly, this study demonstrates that Twist+ CTCs can be utilized as diagnostic and prognostic biomarkers in HCC metastasis.

Although Veridex's CELLSEARCH technology is a standard method used in several studies for isolating CTCs, it is limited in that only epithelial CTCs may be isolated [31]. This could potentially impede clinical application of isolating CTCs from HCC tumors, which are not typical epithelial-type tumors. In our study, the optimized CanPatrol CTC-enrichment technique, which isolates and identifies CTCs by a filter-based method and RNA-ISH technology, was used for CTCs isolation and analysis. Several studies had reported that, compared with other methods, the optimized CanPatrol CTC-enrichment technique is more effective for CTC isolation and characterization [16, 24, 32]. In a study [32], to compare the efficacy of the methods for CTC isolation and characterization, 18 samples were tested by each method. The results showed that a greater number of CTCs were detected in 5 ml of blood by the optimized CanPatrol CTC-enrichment technique. With other methods, some atypical cells were found in samples that were probably unlabeled CTCs. Blood samples were viscous, and the loss of CTCs from these samples by other methods was probably due to the multiple centrifugation and washing steps [24, 33]. Therefore, based on the optimized CanPatrol CTC-enrichment technique, we studied the relationship between the expression of Twist in CTCs and clinicohistopathological risk factors of HCC patients.

We found that the positive ratios of Twist+ CTCs have unique advantages for estimating metastasis and prognosis. The expression of Twist in CTCs can more accurately predict HCC metastasis than CTCs alone. We found that the validity of the optimized CanPatrol CTC-enrichment technique was confirmed by the detection of CTCs from 62 of the 80 (77.5%) HCC patients and by the detection of Twist+ CTCs from 54 of the 80 (67.5%) HCC patients. Firstly, we demonstrated that the positive ratios of CTCs and Twist+ CTCs were significantly higher in HCC patients with portal vein tumor thrombi (*P* = 0.039 and *P* = 0.001), respectively, than in HCC patients without portal vein tumor thrombi. This finding was consistent with the observation that portal vein tumor thrombi have a crucial role in intrahepatic metastasis [34]. It can be assumed that CTCs are the initial stage of portal vein tumor thrombi formation. Compared with the ROC curve for CTCs and Twist+ CTCs in predicting portal vein tumor thrombi, in which the assay demonstrated an AUC of 0.872 and 0.927, respectively, the ROC curve analyses suggested that the prognostic evaluation of Twist+ CTCs outperforms CTCs alone in predicting portal vein tumor thrombi. Furthermore, we also demonstrated that the positive ratios of Twist+ CTCs were closely correlated with TNM staging from stage I to stage IV (*P* = 0.038). The results also indicated that there were a greater proportion of samples containing Twist+ CTCs in the metastatic stages of HCC compared with the earlier stages of HCC (Table 2). Compared with the correlation between the positive ratios of CTCs and TNM staging (*P* = 0.274), the Twist+ CTCs more accurately predicted HCC metastasis (*P* = 0.038). This finding inferred that Twist has a crucial role in enhancing invasiveness and motility by activating EMT in HCC and is consistent with previous reports that Twist overexpression was positively correlated with HCC metastasis [29–35]. Therefore, the close correlation between Twist+ CTCs and tumor number (*P* < 0.001) is understandable. Compared with the ROC curve for CTCs and Twist+ CTCs in predicting tumor number, in which the assay demonstrated an AUC of 0.897 and 0.955, respectively, the ROC curve analyses suggested that the prognostic evaluation of Twist+ CTCs outperforms CTCs alone in predicting tumor number. Our results indicate that there was correlation between the Twist+ CTCs and tumor size (*P* = 0.018), which differed from previous findings. These results do not imply that Twist+ CTCs can predict tumor size. This is probably a consequence of a limited sample size. Interestingly, our results indicate that there was a close correlation between the Twist+ CTCs and cirrhosis (*P* = 0.001). It seems that Twist can account for this hepatic fibrosis. This finding was consistent with previous reports that Twist contributes to proliferation and epithelial-to-mesenchymal transition-induced fibrosis by regulating YB-1 in human peritoneal mesothelial cells [36]. In addition, we demonstrated that the positive ratios of CTCs and Twist+ CTCs were significantly higher in HCC patients with MVI (*P* = 0.052 and *P* = 0.004), respectively, than in HCC patients without MVI. It can be assumed that CTCs are the initial stage of MVI and it can also be inferred that Twist has a crucial role in facilitating EMT in the early phases of HCC progression. However, there were no significant differences between the Twist+ CTCs and age, gender, Child-Pugh class, or tumor differentiation stage.

The follow-up results showed that the positive ratios of Twist+ CTCs were closely correlated with the rate of metastasis or recurrence and the mortality rate of patients who underwent hepatectomy. This study specifically showed that Twist+ CTC was a strong independent prognostic indicator of patients who underwent hepatectomy. However, the difference of PD rate of the patients who underwent TACE was not statistically significant. This result did not imply that Twist+ CTCs could not predict the prognosis of the patients who underwent TACE. This is probably a consequence of a limited sample size.

EMT, accounting for tumor cell plasticity, could initiate the dissemination of CTCs and establish micrometastasis [37]. We checked the expression of epithelial marker (CK19) and mesenchymal marker (Glypican-3) to study the relationship between EMT and Twist; the results showed that the Twist+ CTCs had higher expression in the Glypican-3, which means that the Twist gene may promote the EMT progression by Glypican-3. As reported, the increased expression of Glypican-3 in tumor tissues was closely related to the level of EMT markers and to the cancer vascular invasion [38, 39]. Other studies showed that HepG2 cells, with higher expression of Glypican-3, earned stronger ability of invasion and exhibited more EMT-like changes than those of HCC cell lines that expressed lower levels of Glypican-3 (Hep3B and Huh7)[40–42]. The reason of higher level of metastasis, portal vein tumor thrombi, and tumor number in Twist+ CTC group may be due to EMT progression induced by Glypican-3 [43, 44].

## 5. Conclusion

To our knowledge, this is the first study to investigate the expression of Twist in CTCs of HCC patients with the optimized CanPatrol CTC-enrichment technique. In summary, our study inferred that transcription factor Twist might facilitate EMT in the early phases of HCC progression, and Twist+ CTCs may potentially serve as a prognostic indicator that has unique advantages compared with other traditional diagnostic methods. We posit that detection of Twist+ CTCs could help clinicians to better characterize cancer cases and exploit better therapeutic strategies to improve the prognosis of HCC patients based on prognosis monitoring. Furthermore, we observed a correlation between Twist+ CTCs and cirrhosis for the first time. It implies that Twist+ CTCs may be used for estimation of cirrhosis. The reason of higher level of metastasis, portal vein tumor thrombi, and tumor number in Twist+ CTC group may be due to EMT progression induced by Glypican-3. However, we must point out that there is a limitation in our study. The limited sample size might have impacted our conclusions. It would be meaningful to follow up with large-scale trials of HCC patients at different stages of treatment. Long-term follow-up studies are essential to understand how Twist+ CTCs could be used to predict relapse and metastasis.

## Figures and Tables

**Table 1:** Information and clinical characteristics of the 80 HCC patients.

**Clinical variables**	**Number (%)**	**Number of samples containing CTCs (%)**	**Number of samples containing Twist+ CTCs (%)**	**CTCs numbers**		**Twist+ CTCs numbers**	
				**Range**	**Average**	**Range**	**Average**
**Age (years)**							
<30	19 (23.70%)	7 (36.84%)	6 (31.58%)	0~49	13.93	0~12	3.65
30-50	39 (48.80%)	34 (87.18%)	29 (74.36%)	2~52	14.37	0~16	3.87
>50	22 (27.50%)	21 (95.45%)	19 (86.37%)	1~50	14.07	0~13	3.19
**Gender**							
Males	70 (87.50%)	55 (78.57%)	48 (68.57%)	0~52	14.41	0~16	4.05
Females	10 (12.50%)	7 (70.00%)	6 (60.00%)	0~49	14.04	0~14	3.55
**Child-Pugh class**							
A	62 (77.50%)	44 (70.97%)	36 (58.06%)	0~48	14.04	0~12	2.96
B	12 (15.00%)	12 (100.00%)	12 (100.00%)	1~35	14.71	0~13	3.57
C	6 (7.50%)	6 (100.00%)	6 (100.00%)	4~52	15.22	1~16	4.68
**Cirrhosis**							
With	57 (71.25%)	52 (91.23%)	51 (89.47%)	0~52	14.84	0~16	3.46
Without	23 (28.75%)	10 (43.48%)	3 (13.04%)	0~50	14.17	0~13	3.19
**Tumor number**							
Single	31 (38.75%)	14 (45.16%)	6 (19.35%)	0~38	12.88	0~12	2.55
Multiple	49 (61.25%)	48 (97.96%)	48 (97.96%)	1~52	17.05	0~16	4.27
**Tumor size(cm)**							
<3	16 (20.00%)	5 (31.25%)	2 (12.50%)	0~24	13.02	0~11	2.84
3-5	19 (23.80%)	15 (78.95%)	12 (63.16%)	0~44	17.47	0~16	3.41
>5	45 (56.20%)	42 (93.30%)	40 (88.89%)	2~52	20.05	1~16	4.51
**AFP (ng/ml)**							
<20	17 (21.25%)	6 (35.29%)	3 (17.65%)	0~50	14.15	0~12	3.97
20-100	19 (23.75%)	13 (68.42%)	9 (47.37%)	0~51	15	0~13	3.47
100-500	14 (17.50%)	14 (100.00%)	14 (100.00%)	0~50	14.53	0~16	3.41
>500	30 (37.50%)	29 (96.67%)	28 (93.33%)	0~52	15.13	0~14	3.59
**Portal vein tumor thrombus**							
With	46 (57.50%)	46 (100.00%)	46 (100.00%)	3~52	25.57	1~16	5.26
Without	34 (42.50%)	16 (47.06%)	8 (23.53%)	0~37	14.42	0~12	2.14
**∗** **TNM staging**							
Stage I	9 (11.25%)	2 (22.22%)	0 (00.00%)	0~33	13.62	0~11	2.02
Stage II	25 (31.25%)	17 (68.00%)	13 (52.00%)	0~36	16.03	0~13	2.44
Stage III	36 (45.00%)	33 (91.67%)	31 (86.11%)	0~50	21.47	1~16	4.64
Stage IV	10 (12.50%)	10 (100.00%)	10 (100.00%)	2~52	25.17	1~16	5.82

**Note.**  *∗*American Joint Committee on Cancer TNM Staging for Liver Tumors (7th edition, 2010, ISBN 0387884408).

AFP: alpha-fetoprotein; HCC: hepatocellular carcinoma; CTCs: circulating tumor cells.

**Table 2:** Correlation between the positive ratios of CTCs/Twist+ CTCs and clinicohistopathological risk factors in the 80 HCC patients.

Clinical variable	CTCs		Twist+ CTCs	
	*χ* ^2^	*P value*	*χ* ^2^	*P value*
Age	3.682	0.159	3.539	0.17
Gender	0.049	0.826	0.059	0.808
Cirrhosis	3.119	0.077	11.091	0.001^b^
AFP	4.188	0.242	8.724	0.033^a^
Child-Pugh class	0.788	0.674	1.926	0.382
Tumor number	4.219	0.040^a^	12.321	<0.001^b^
Tumor size	4.120	0.127	8.021	0.018^a^
Portal vein tumor thrombus	4.267	0.039^a^	11.483	0.001^b^
*∗*TNM staging	3.890	0.274	8.435	0.038^a^

**Note.**  *P* values are from chi-squared test or Fisher's exact test. ^a^Correlation is significant at the 0.05 level (2-tailed). ^b^Correlation is significant at the 0.001 level (2-tailed). *∗*American Joint Committee on Cancer TNM Staging for Liver Tumors (7th edition, 2010, ISBN 0387884408).

AFP: alpha-fetoprotein; HCC: hepatocellular carcinoma; CTCs: circulating tumor cells.

**Table 3:** Correlation between the positive ratios of CTCs/Twist+ CTCs and histopathological risk factors of the 41 HCC patients who underwent hepatectomy.

Histopathological features	CTCs		Twist+ CTCs	
	*χ* ^2^	*P value*	*χ* ^2^	*P value*
*∗*Differentiation stage	3.425	0.232	4.909	0.089
Microvascular invasion	4.143	0.052	9.616	0.004^a^

**Note.**  *P* values are from chi-squared test or Fisher's exact test. ^a^Correlation is significant at the 0.05 level (2-tailed). *∗*Edmondson-Steiner grading system.

HCC: hepatocellular carcinoma; CTCs: circulating tumor cells.

**Table 4:** Glypican-3 protein expression in the samples of the 41 HCC patients who underwent hepatectomy.

Classification	Patient number	Glypican-3 (+)	Glypican-3 (-)	*χ* ^2^	*P* value
CTC+ group	28	19	9	1.757	0.185
CTC- group	13	6	7		

Twist+ CTC group	24	19	5	8.050	0.005^a^
Twist- CTC group	17	6	11		

**Note.**   *P* values are from chi-squared test or Fisher's exact test. ^a^Correlation is significant at the 0.05 level (2-tailed).

HCC: hepatocellular carcinoma; CTCs: circulating tumor cells.

**Table 5:** The prognosis of the 33 patients who underwent hepatectomy.

Classification	Patient number	Number of metastases/recurrences	Number of no recurrences and no metastases	*χ* ^2^	*P* value	Number of deaths	Number of survival cases	*χ* ^2^	*P* value
CTC+ group	25	20	5	5.183	0.036^a^	19	6	6.812	0.015^a^
CTC- group	8	3	5			2	6		

Twist+ CTC group	21	19	2	11.806	0.001^b^	18	3	12.165	0.001^b^
Twist- CTC group	12	4	8			3	9		

**Note.** 33 HCC patients underwent hepatectomy. All patients were followed up for no less than 1 year. *P* values are from Fisher's exact test. ^a^Correlation is significant at the 0.05 level (2-tailed). ^b^Correlation is significant at the 0.001 level (2-tailed).

CTCs: circulating tumor cells; HCC: hepatocellular carcinoma.

**Table 6:** The prognosis of the 14 patients who underwent TACE.

Classification	Patients number	Number of CR+PR+SD	Number of PD	*χ* ^2^	*P* value	Number of deaths	Number of survival cases	*χ* ^2^	*P* value
CTC+ group	9	2	7	1.998	0.266	6	3	0.933	0.580
CTC- group	5	3	2			2	3		

Twist+ CTC group	8	1	7	4.381	0.091	6	2	2.431	0.277
Twist- CTC group	6	4	2			2	4		

**Note.** 14 HCC patients underwent TACE. All patients were followed up for no less than 1 year. *P* values are from Fisher's exact test.

CTCs: circulating tumor cells; HCC: hepatocellular carcinoma; TACE: transcatheter arterial chemoembolization; CR: complete response; PR: partial response; SD: stable disease; PD: progressive disease.

## Data Availability

The data used to support the findings of this study are included within the article and the supplementary material files.

## Supplementary Materials

Supplementary 1 Assessment of TACE: mRECIST assessment for HCC following the AASLD-JNCI Guideline.

Supplementary 2 Capture probe sequences for the EpCAM, CK8/18/19, Twist, and CD45 genes.

Supplementary 3 EpCAM, CK8/18/19, and Twist expression in HepG2 tumor cells and leukocytes.

Supplementary 4 Histopathological features of the 41 HCC patients who underwent hepatectomy.

Supplementary 5 Number variation of CTCs and Twist+ CTCs after HCC therapy.
