# Supplementary material for: Twist Expression in Circulating Hepatocellular Carcinoma Cells Predicts Metastasis and Prognoses
Source: Biomed Res Int. 2018 Jun 26;2018:3789613. doi: 10.1155/2018/3789613 (PMC6038670; doi:10.1155/2018/3789613)
Supplement: Supplementary 1 — Assessment of TACE: mRECIST assessment for HCC following the AASLD-JNCI Guideline. [file 3789613.f1.docx]

| Supplementary 1**:** Assessment of TACE: mRECIST Assessment for HCC Following the AASLD-JNCI Guideline |
| --- |
|  |
| mRECIST criteria |
| CR: Disappearance of any intratumoral arterial enhancement in all target lesions. |
| PR: At least a 30% decrease in the sum of diameters of viable (enhancement in the arterial phase) target lesions, taking as reference the baseline sum of the diameters of target lesions. |
|  |
|  |
| SD: Any cases that do not qualify for either partial response or progressive disease. |
| PD: An increase of at least 20% in the sum of the diameters of viable(enhancing) target lesions, taking as reference the smallest sum of the diameters of viable(enhancing) target lesions recorded since treatment started. |
|  |
|  |

**Notes:** AASLD: American Association for the Study of Liver Diseases; JNCI: Joumal of the National Cancer Institute; HCC: Hepatocellular carcinoma; mRECIST: modified Response Evaluation Criteria in Solid Tumors; CR: Complete response; PR: Partial response; SD: Stable disease; PD: Progressive disease.
