# Supplementary material for: Twist Expression in Circulating Hepatocellular Carcinoma Cells Predicts Metastasis and Prognoses
Source: Biomed Res Int. 2018 Jun 26;2018:3789613. doi: 10.1155/2018/3789613 (PMC6038670; doi:10.1155/2018/3789613)
Supplement: Supplementary 2 — Capture probe sequences for the EpCAM, CK8/18/19, Twist, and CD45 genes. [file 3789613.f2.docx]

Supplementary 2**:** Capture probe sequences for the EpCAM, CK8/18/19, Twist, and CD45 genes.

| **Gene** |  |  |  |  | **Sequences(5'→3')** |
| --- | --- | --- | --- | --- | --- |
| EpCAM |  |  |  |  | TGGTGCTCGTTGATGAGTCA |
|  |  |  |  |  | AGCCAGCTTTGAGCAAATGA |
|  |  |  |  |  | AAAGCCCATCATTGTTCTGG |
|  |  |  |  |  | CTCTCATCGCAGTCAGGATC |
|  |  |  |  |  | TCCTTGTCTGTTCTTCTGAC |
|  |  |  |  |  | CTCAGAGCAGGTTATTTCAG |
| CK8 |  |  |  |  | CGTACCTTGTCTATGAAGGA |
|  |  |  |  |  | ACTTGGTCTCCAGCATCTTG |
|  |  |  |  |  | CCTAAGGTTGTTGATGTAGC |
|  |  |  |  |  | CTGAGGAAGTTGATCTCGTC |
|  |  |  |  |  | CAGATGTGTCCGAGATCTGG |
|  |  |  |  |  | TGACCTCAGCAATGATGCTG |
| CK18 |  |  |  |  | AGAAAGGACAGGACTCAGGC |
|  |  |  |  |  | GAGTGGTGAAGCTCATGCTG |
|  |  |  |  |  | TCAGGTCCTCGATGATCTTG |
|  |  |  |  |  | CAATCTGCAGAACGATGCGG |
|  |  |  |  |  | AAGTCATCAGCAGCAAGACG |
|  |  |  |  |  | CTGCAGTCGTGTGATATTGG |
| CK19 |  |  |  |  | CTGTAGGAAGTCATGGCGAG |
|  |  |  |  |  | AAGTCATCTGCAGCCAGACG |
|  |  |  |  |  | CTGTTCCGTCTCAAACTTGG |
|  |  |  |  |  | TTCTTCTTCAGGTAGGCCAG |
|  |  |  |  |  | CTCAGCGTACTGATTTCCTC |
|  |  |  |  |  | GTGAACCAGGCTTCAGCATC |
| Twist |  |  |  |  | ACAATGACATCTAGGTCTCC |
|  |  |  |  |  | CTGGTAGAGGAAGTCGATGT |
|  |  |  |  |  | CAACTGTTCAGACTTCTATC |
|  |  |  |  |  | CCTCTTGAGAATGCATGCAT |
|  |  |  |  |  | TTTCAGTGGCTGATTGGCAC |
|  |  |  |  |  | TTACCATGGGTCCTCAATAA |
| CD45 |  |  |  |  | TCGCAATTCTTATGCGACTC |
|  |  |  |  |  | TGTCATGGAGACAGTCATGT |
|  |  |  |  |  | GTATTTCCAGCTTCAACTTC |
|  |  |  |  |  | CCATCAATATAGCTGGCATT |
|  |  |  |  |  | TTGTGCAGCAATGTATTTCC |
|  |  |  |  |  | TACTTGAACCATCAGGCATC |

EpCAM: epithelial cell adhesion molecule; CK: cytokeratins.
