# Supplementary material for: Twist Expression in Circulating Hepatocellular Carcinoma Cells Predicts Metastasis and Prognoses
Source: Biomed Res Int. 2018 Jun 26;2018:3789613. doi: 10.1155/2018/3789613 (PMC6038670; doi:10.1155/2018/3789613)
Supplement: Supplementary 3 — EpCAM, CK8/18/19, and Twist expression in HepG2 tumor cells and leukocytes. [file 3789613.f3.docx]

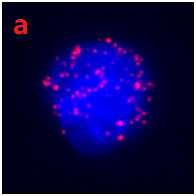

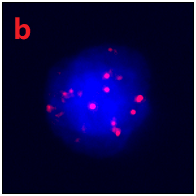

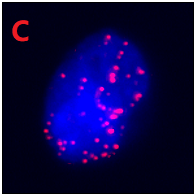

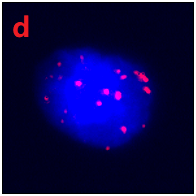

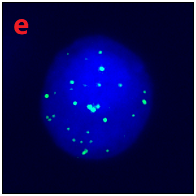

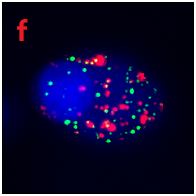

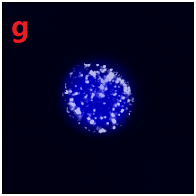


Supplementary 3**:** EpCAM, CK8/18/19, and Twist expression in HepG2 tumor cells and leukocytes.

**Notes:** (a): HepG2 cells stained for EpCAM expression (red fluorescence); (b): HepG2 cells stained for CK8 expression (red fluorescence); (c): HepG2 cells stained for CK18 expression (red fluorescence); (d): HepG2 cells stained for CK19 expression (red fluorescence); (e): HepG2 cells stained for Twist expression (green fluorescence); (f): HepG2 cells stained for EpCAM, CK8/18/19, and Twist expression (red/green fluorescence); (g): negative control, leukocytes stained for CD45 expression (bright white fluorescence); The cells were analyzed using a 100x oil objective.

HepG2: human hepatocarcinoma; EpCAM: epithelial cell adhesion molecule; CK: cytokeratin.
