# Supplementary material for: Twist Expression in Circulating Hepatocellular Carcinoma Cells Predicts Metastasis and Prognoses
Source: Biomed Res Int. 2018 Jun 26;2018:3789613. doi: 10.1155/2018/3789613 (PMC6038670; doi:10.1155/2018/3789613)
Supplement: Supplementary 4 — Histopathological features of the 41 HCC patients who underwent hepatectomy. [file 3789613.f4.docx]

| Supplementary 4**:** Histopathologic features of the 41 HCC patients who underwent hepatectomy | | | | | | | |
| --- | --- | --- | --- | --- | --- | --- | --- |
| **Histopathologic features** | **Number(%)** | **Number of samples containing CTCs (%)** | **Number of samples containing Twist+ CTCs(%)** | **CTCs numbers** | | **Twist+ CTCs numbers** | |
|  |  |  |  | **Range** | **Average** | **Range** | **Average** |
| ***Differentiation stage** |  |  |  |  |  |  |  |
| High(I) | 14(34.15%) | 7(50.00%) | 5(35.71%) | 0～41 | 13.13 | 0～9 | 3.19 |
| Middle(II+III) | 16(39.02%) | 12(75.00%) | 12(75.00%) | 1～44 | 14.37 | 0～13 | 3.52 |
| Low(IV) | 11(26.83%) | 9(81.82%) | 7(63.64%) | 3～52 | 15.23 | 2～16 | 4.04 |
| **Microvascular invasion** |  |  |  |  |  |  |  |
| With | 19(46.34%) | 16(84.21%) | 16(84.21%) | 0～52 | 14.37 | 1～16 | 4.55 |
| Without | 22(53.66%) | 12(54.55%) | 8(36.36%) | 0～41 | 11.04 | 0～12 | 3.07 |

**Notes:** *Edmondson-Steiner grading system.

HCC: hepatocellular; CTCs: circulating tumor cells.
