# Supplementary material for: Twist Expression in Circulating Hepatocellular Carcinoma Cells Predicts Metastasis and Prognoses
Source: Biomed Res Int. 2018 Jun 26;2018:3789613. doi: 10.1155/2018/3789613 (PMC6038670; doi:10.1155/2018/3789613)
Supplement: Supplementary 5 — Number variation of CTCs and Twist+ CTCs after HCC therapy. [file 3789613.f5.docx]

**(b)**

**(a)**

**(d)**

**(c)**

Supplementary 5**:** Number variation of CTCs and Twist+ CTCs after HCC therapy.

**Notes:** CTCs and Twist+ CTCs were collected and analyzed from the same patients before and after therapy. (a) CTCs and Twist+ CTCs were abtained from the stage I HCC patient (NO.51) with hepatectomy; (b) CTCs and Twist+ CTCs were abtained from the stage II HCC patient (NO.69) with hepatectomy; (c) CTCs and Twist+ CTCs were abtained from the stage III HCC patient (NO.21) with hepatectomy; (d) CTCs and Twist+ CTCs were abtained from the stage IV HCC patient (NO.29) with TACE.

CTCs: circulating tumor cells; HCC: Hepatocellular carcinoma; TACE: transcatheter arterial chemoembolization.
